# Targeting triple-negative breast cancer cells with a β1-integrin binding aptamer

**DOI:** 10.1016/j.omtn.2023.08.015

**Published:** 2023-08-16

**Authors:** Karlis Pleiko, Maarja Haugas, Vadims Parfejevs, Teodors Pantelejevs, Emilio Parisini, Tambet Teesalu, Una Riekstina

**Affiliations:** 1 Faculty of Medicine, University of Latvia, House of Science, Jelgavas Str. 3, 1004 Riga, Latvia; 2 Laboratory of Precision- and Nanomedicine, Institute of Biomedicine and Translational Medicine, University of Tartu, 50411 Tartu, Estonia; 3 Latvian Institute of Organic Synthesis, Aizkraukles 21, 1006 Riga, Latvia; 4 Department of Chemistry “G. Ciamician”, University of Bologna, Via Selmi 2, 40126 Bologna, Italy; 5 Materials Research Laboratory, University of California, Santa Barbara, Santa Barbara, CA 93106, USA

**Keywords:** MT: Oligonucleotides: Therapies and Applications, aptamer, β1-integrin, aptamer internalization, proximity labeling, affinity ligands, triple-negative breast cancer

## Abstract

Targeted therapies have increased the treatment options for triple-negative breast cancer patients. However, the paucity of targetable biomarkers and tumor heterogeneity have limited the ability of precision-guided interventions to live up to their full potential. As affinity-targeting ligands, aptamers show high selectivity toward target molecules. Compared with antibodies, aptamers have lower molecular weight, increased stability during transportation, reduced immunogenicity, and increased tissue uptake. Recently, we reported discovery of the GreenB1 aptamer, which is internalized in cultured triple-negative MDA-MB-231 human breast cancer cells. We show that the GreenB1 aptamer specifically targets β1-integrin, a protein linked previously to breast cancer cell invasiveness and migration. Aptamer binds to β1-integrin with low nanomolar affinity. Our findings suggest potential applications for GreenB1-guided precision agents for diagnosis and therapy of cancers overexpressing β1-integrin.

## Graphical abstract

**Figure:**
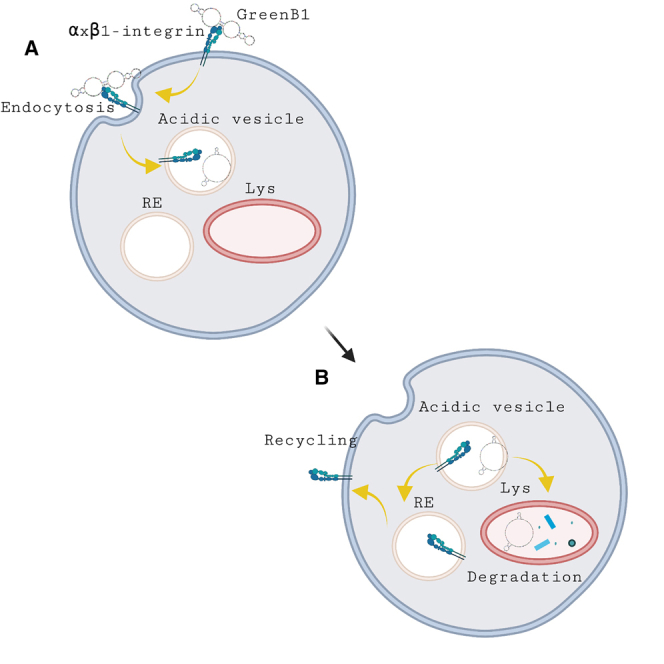

## Teaser

Pleiko and colleagues have discovered a GreenB1 aptamer that specifically targets a cell-surface protein linked to cancer cell invasiveness and migration. The aptamer demonstrates low nanomolar affinity for the protein and could be used as a diagnostic or therapeutic agent for cancers with high levels of the protein.

## Introduction

Triple-negative breast cancer (TNBC) accounts for ∼20% of all invasive breast cancer cases. TNBC tumors are negative for expression of human epidermal growth factor receptor 2 (HER-2), progesterone receptor (PR), and estrogen receptor 2 (ER-beta), rendering TNBC resistant to endocrine therapy.^1^ Chemotherapy followed by surgery is used as a treatment strategy for early TNBC, while chemotherapy is used to treat advanced and metastatic TNBC.^2^ Treatments with immune checkpoint inhibitors (ICIs) targeting programmed cell death protein 1 (PD-1) and programmed death ligand 1 (PD-L1) have augmented the therapeutic choices for patients with PD-L1^+^ TNBC in recent years.^3^

Appreciation of the heterogeneity of the TNBC microenvironment (TME), including differences in immunological composition, vascularization, metabolic status, and stromal composition, has resulted in identifying TNBC subtypes with different treatment responses.^4^ TNBC has at least three subtypes (basal, luminal androgen receptor, and mesenchymal). Single-cell sequencing has revealed tumor microenvironment heterogeneity, showing populations of cells typical of cancers with poor outcomes^5^ and subtypes based on gene-regulatory networks.^6^ Development of therapies that target each subtype may increase the number of available treatment options in the future.^7^

Based on TME differences, tumors can be divided into “hot” tumors, a T cell-inflamed cancer phenotype, and “cold” tumors, a non-T cell-inflamed phenotype. Current ICIs are limited to acting on “hot” tumors.^8^ Anticancer vaccines, targeted therapies that increase re-expression of tumor-associated antigens, engineered T cells expressing chimeric antigen receptors (CARs), and other^9^ approaches are being studied to promote T cell infiltration, transforming “cold” tumors into ICI-responsive hot tumors. Furthermore, targeted therapy is used against TME cellular components; for example, OximUNO (a nanoconjugate of CD206 targeting peptide mUNO with doxorubicin) has shown promise in pre-clinical studies to inhibit breast cancer progression by depleting anti-inflammatory, tumor-supporting macrophages.^10^

Antibody-drug conjugates (ADCs) have been used successfully as guided precision agents.^11^ One such ADC, sacituzumab govitecan, composed of antibody targeting trophoblast cell-surface antigen 2 (TROP2) linked to SN-38 (topoisomerase I inhibitor) through a hydrolyzable linker has received US Food and Drug Administration (FDA) approval for treatment of metastatic TNBC.^12^ Several other ADCs are undergoing clinical trials and have been reviewed recently.^13^ The bispecific antibody PF-06671008, which targets CD3 on T cells and P-cadherin (CDH3) on tumor cells, is another promising strategy for T cell recruitment to tumor sites.^14^ It has been investigated in a phase I clinical trial (ClinicalTrials.gov: NCT02659631) for treatment of advanced solid tumors. However, present treatments do not yet provide optimal therapy options.

Aptamers are short (20–100 nt), single-stranded DNA or RNA oligonucleotides that bind to their target molecules because of a specific three-dimensional structure. Their affinity and specificity are comparable with antibodies; however, aptamers are smaller (6–30 kDa versus 150–180 kDa for antibodies) and can be chemically synthesized, resulting in minimal to no batch-to-batch variability and straightforward scale up. Aptamers are stable, can be denatured/refolded, and have rapid tissue uptake^15^ and low immunogenicity.^16^ Recently, Kelly et al.^17^ have highlighted considerable difficulties when translating aptamers selected under cell-free settings to *in vitro* and *in vivo* studies. Of the 15 aptamers that were reported to target cell surface proteins, 5 showed receptor-specific activity on cells *in vitro*. Of the three aptamers that were tested in animals, only one (Waz) was able to target tumors *in vivo*. Two other aptamers, E07min and Sgc8c, had already been tested previously *in vivo*.^17^

Target-specific aptamers have been utilized to create tools for detecting circulating targets (circulating tumor cells, proteins, extracellular vesicles),^18^^,^^19^ aptamer-targeted vesicles or nanoparticles that improve medication delivery,^20^^,^^21^ and fluorescent RNA-based biosensors for metabolite detection.^22^

Selection on TNBC-related proteins or on cultured TNBC cells has identified multiple aptamers. Epidermal growth factor receptor (EGFR),^23^^,^^24^^,^^25^^,^^26^ platelet-derived growth factor receptor β (PDGFRB),^27^^,^^28^^,^^29^ nucleolin (NCL),^30^^,^^31^^,^^32^^,^^33^^,^^34^^,^^35^^,^^36^ CD133,^37^ CD44,^38^^,^^39^ epithelial cell adhesion molecule (EpCAM),^40^^,^^41^ CD49c,^42^ and Tenascin-C (TNC)^43^ binding aptamers have shown potential for selective delivery of therapeutic agents to TNBC *in vitro* and *in vivo*.

Here we show that the TNBC cell line-selective aptamer GreenB1 binds to β1-integrins and is internalized in cells.

## Results

### GreenB1 binds to cultured TNBC cells

**Figure 1:**
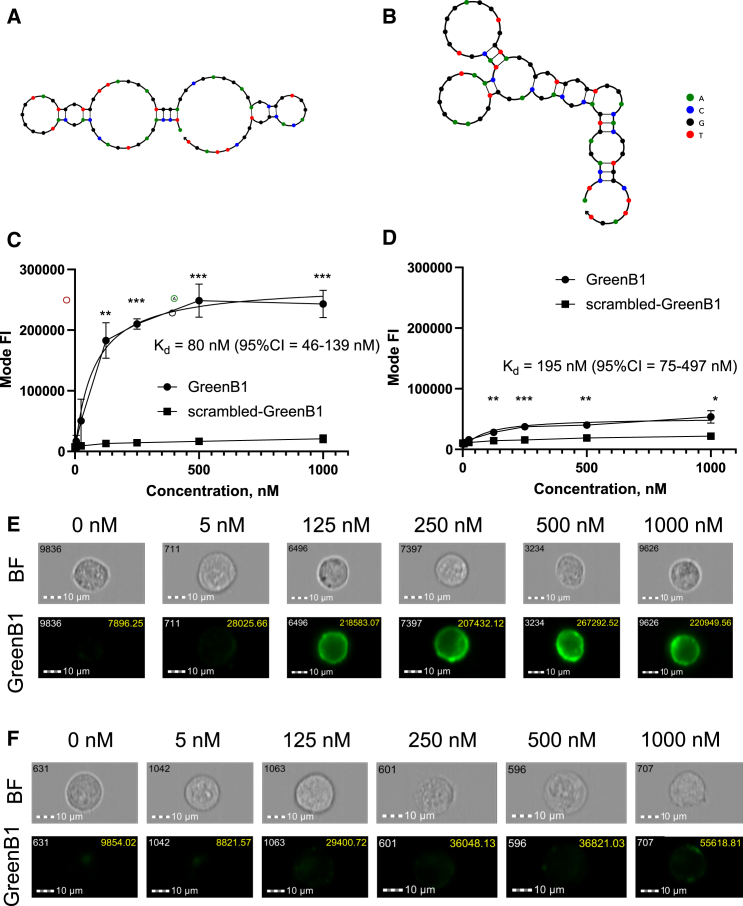
GreenB1 binds to TNBC cells (A and B) GreenB1 (A) and scr-GreenB1 (B) secondary structure as predicted by NUPACK software. (C) GreenB1 and scr-GreenB1 binding to the MDA-MB-231 TNBC cell line at 0, 5, 25, 125, 250, 500, and 1,000 nM concentrations. (D) GreenB1 and scr-GreenB1 binding to the MCF-7 PR- and ER-expressing cell line at 0, 5, 25, 125, 250, 500, and 1,000 nM concentrations. (E and F) Imaging flow cytometry images of GreenB1 binding to the MDA-MB-231 (E) and MCF-7 (F) cell lines at different concentrations. The values in the top right corner of the FAM channel image show the fluorescence intensities of the whole cells. ∗p < 0.05, ∗∗p < 0.01, ∗∗∗p < 0.001. Error bars indicate SD.

The GreenB1 aptamer was originally identified by us in a SELEX (Systematic Evolution of Ligands by EXponential enrichment) on cultured malignant cells.^44^ Although GreenB1 was identified in a screen for clear cell renal cell carcinoma cell line binders, it also selectively bound the MDA-MB-231 breast cancer cell line. The 6-carboxyfluorescein (FAM)-labeled GreenB1 aptamer (Figure 1A) or FAM-labeled scrambled version of GreenB1 (scrambled GreenB1 [scr-GreenB1]) (Figure 1B) were incubated with two human TNBC cell lines, MDA-MB-231 and MDA-MB-436, and the PR- and ER-positive human breast cancer cell line MCF-7. Incubation was performed in the presence of increasing concentrations (5–1,000 nM) of either GreenB1 or scr-GreenB1 on ice for 1 h. Cell-bound fluorescence was analyzed using imaging flow cytometry. GreenB1 resulted in statistically significantly increased fluorescence intensity compared with scr-GreenB1 at 125 nM and 25 nM concentrations after incubation with the TNBC cell lines MDA-MB-231 (Figures 1C and S1) and MDA-MB-436 (Figures S2 and S3), respectively. However, a statistically significant but only moderate fluorescence increase was observed when incubated with the MCF-7 cell line (Figures 1D and S4). These results suggest that GreenB1 binds selectively to surface protein expressed on cultured TNBC cells.

### Proximity labeling identifies β1-integrin as the target protein

**Figure 2:**
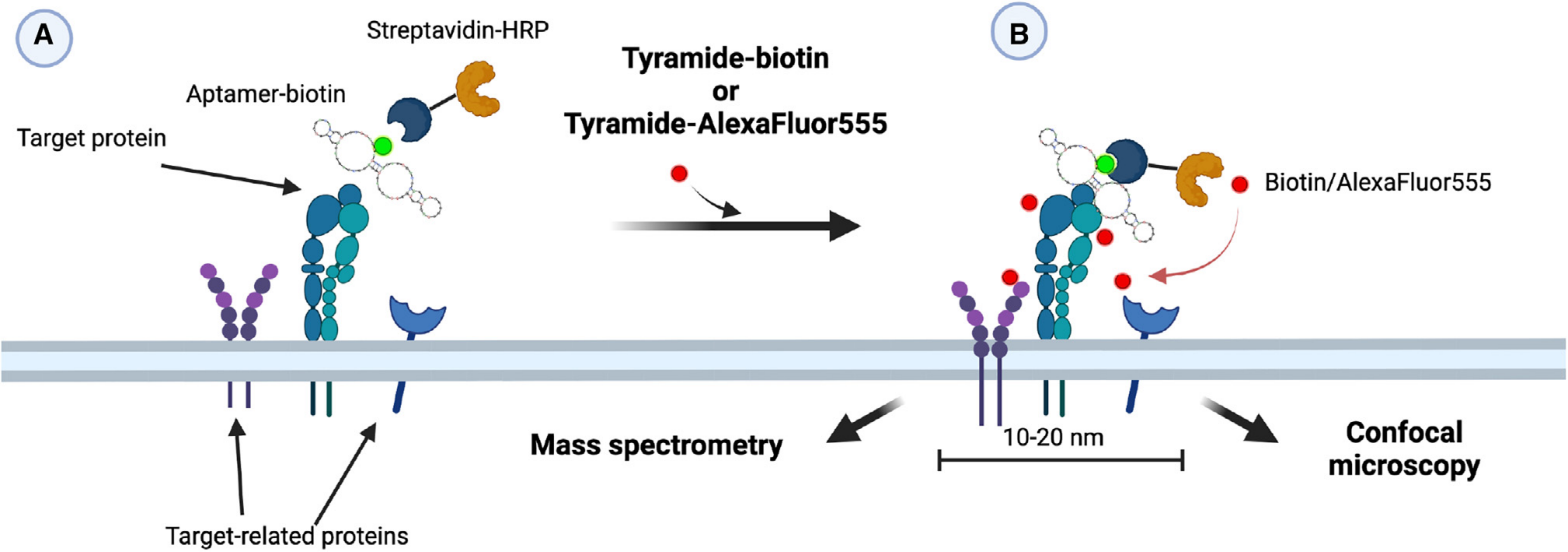
GreenB1 protein target identification using proximity labeling Biotin-labeled GreenB1 or RND was incubated with horseradish peroxidase (HRP) conjugated to streptavidin. (A) Complexes or streptavidin-HRP alone were incubated with live MDA-MB-231 cells for 1 h. After washing away the unbound complex, tyramide-biotin or tyramide-Alexa Fluor 555 with hydrogen peroxide was added to cells for 2 min. (B) HRP, in the presence of hydrogen peroxide, creates a highly reactive tyramide species that labels nearby proteins. Fluorescently labeled proteins were further imaged using confocal microscopy. Biotinylated proteins were pulled down using streptavidin-coated magnetic beads, eluted using 25 mM biotin in lysis buffer, and heated at 95°C for 5 min. Eluates were run on the gel and analyzed using MS.

The GreenB1 target protein was identified by a proximity ligation-based approach (Figure 2). GreenB1-biotin or random aptamer library (RND)-biotin were complexed with streptavidin-horseradish peroxidase (SA-HRP) and incubated with live MDA-MB-231 cells, followed by a proximity labeling reaction using tyramide-Alexa Fluor 555 or tyramide-biotin.

**Figure 3:**
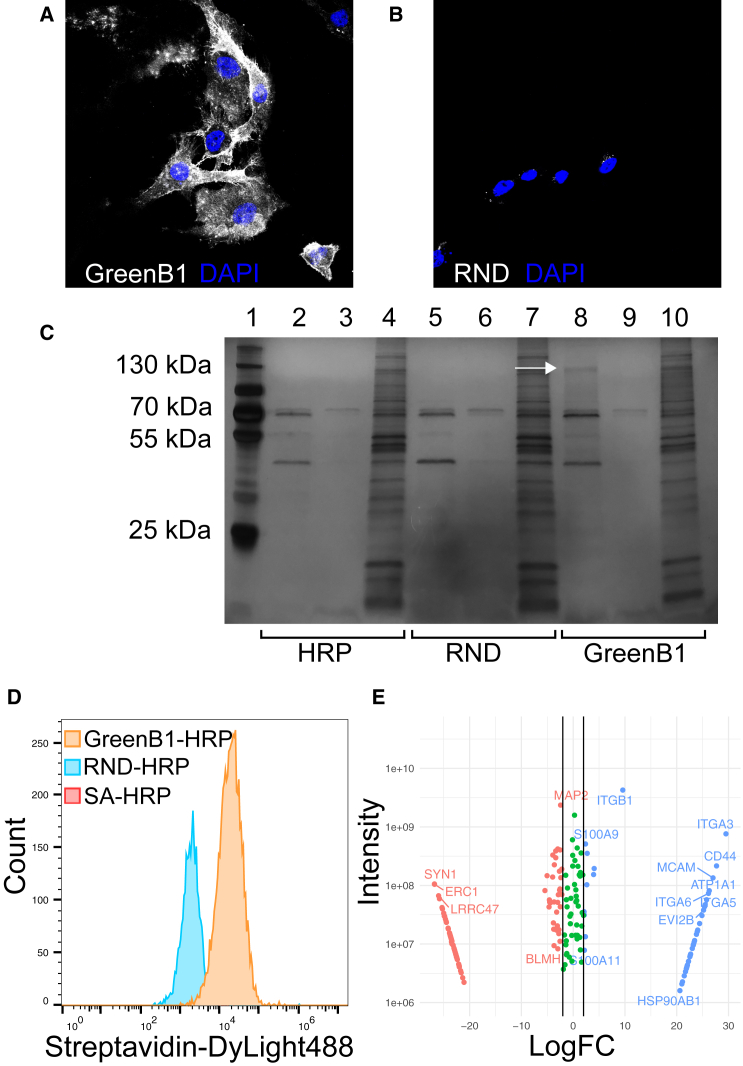
Proximity labeling results for GreenB1 target identification (A and B) Confocal microscopy images of proximity labeling using tyramide-Alexa Fluor 555 with MDA-MB-231 cells after binding of the GreenB1-HRP complex (A) or RND-HRP complex (B). (C) Pull-down results from proximity labeling with tyramide-biotin. Lane 1 contains a marker. Streptavidin-HRP (lanes 2, 3, and 4), RND-HRP (lanes 5, 6, and 7), and GreenB1-HRP (lanes 8, 9, and 10). The first two lanes in each sample were eluted using biotin and heat; the third was eluted using reducing sample buffer. (D) Flow cytometry of biotinylated MDA-MB-231 cells labeled with Streptavidin-DyLight488. (E) MS proteomics of control and target bands corresponding to 130 kDa Log2FC is shown on the x axis. Combined signal intensity from both samples is shown on the y axis.

Reaction of the GreenB1-HRP complex with tyramide-Alexa Fluor 555 on MDA-MB-231 cells resulted in staining observable under a confocal microscope (Figure 3A) with a much higher intensity than when using the RND complex (Figure 3B).

Biotinylated proteins were pulled down using streptavidin-coated magnetic beads. A single band of ∼130 kDa was observed in the first eluate of the GreenB1 sample (Figure 3C, lane 8) but not in RND (Figure 3C, lane 5) or streptavidin-HRP alone (Figure 3C, lane 2) samples. The region containing the band (Figure 3C, indicated with a white arrow) in the GreenB1 sample and the corresponding molecular weight region from the RND sample were subjected to mass spectrometry (MS) proteomics analysis. Flow cytometry confirmed higher labeling of GreenB1-HRP samples compared with RND-HRP or SA-HRP alone (Figure 3D). From MS proteomics data, after filtering out contaminant proteins, keeping proteins with at least 2 unique peptides and proteins with a signal intensity ratio in the GreenB1 sample over the RND sample of at least 10, we identified 28 proteins (Table S1). Three proteins with the highest MS intensity and highest logarithmic fold change (logFC) difference between RND and GreenB1 samples were β1-and α3-integrin and CD44 (Figure 3E). The molecular weight of β1-integrin (around 120–130 kDa)^45^ on SDS-PAGE and the location of the target band further supported β1-integrin being the target protein for the GreenB1 aptamer.

### GreenB1 has low nanomolar affinity for β1-integrin

**Figure 4:**
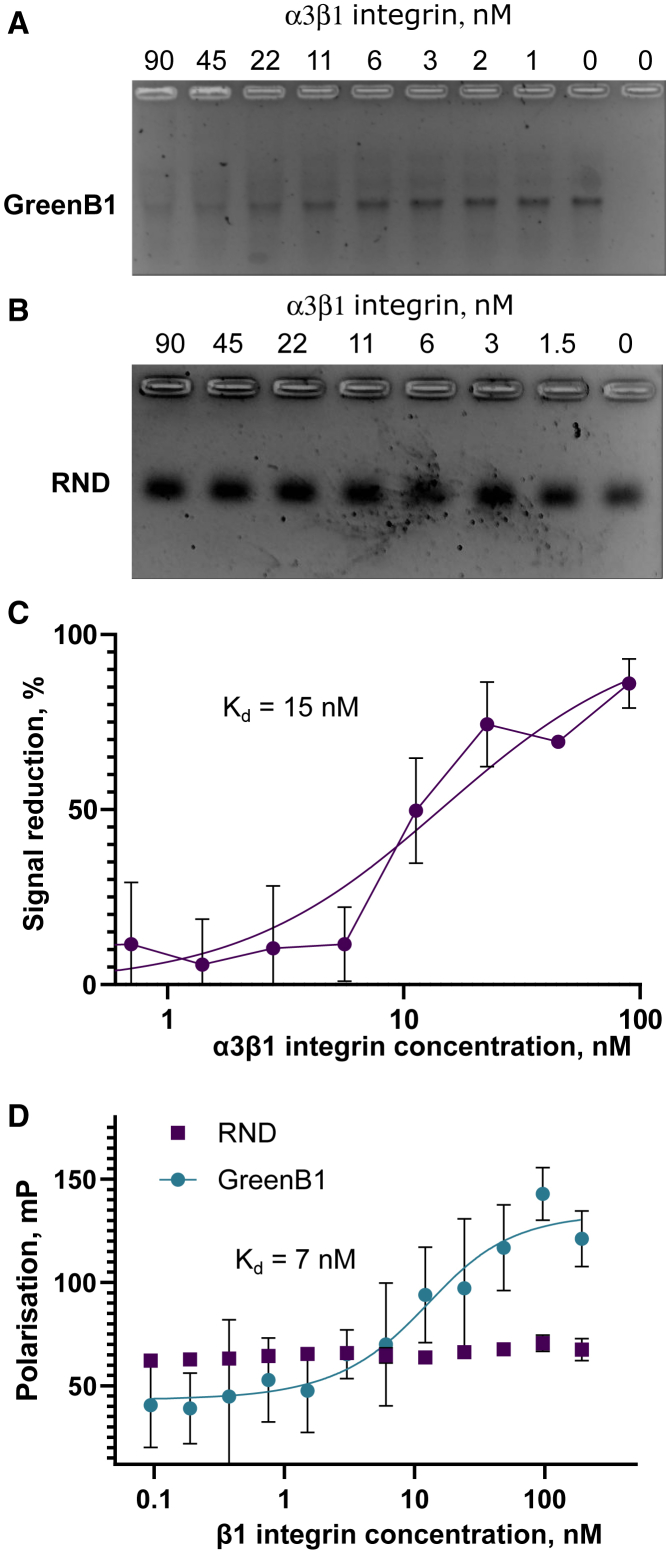
GreenB1 binding to the α3β1-complex and β1-integrin (A and B) Electrophoretic mobility shift assay (EMSA) using increasing concentrations of α3β1-integrin protein at 17.5 nM fixed concentration of GreenB1 (A) or RND (B) aptamers. (C) GreenB1 and α3β1-integrin EMSA results plotted as a reduction of GreenB1 signal intensity (K_D_ = 15 nM, 95% CI 8–26 nM). (D) FP using 10 nM FAM-labeled GreenB1 aptamer or FAM-labeled RND and varying concentrations of β1-integrin (K_D_ = 7 nM, 95% CI 0–17 nM). Plots depict averages of triplicate measurements ± SE and the fitted model.

MS resulted in several additional hits besides α3- and β1-integrin and CD44 (Table S1). Because β1-integrin had a much higher signal intensity than α3-integrin in MS proteomics results, it could be the target protein within the α3β1-integrin complex. To confirm binding and determine the dissociation constant (K_D_) of GreenB1 for α3β1- and β1-integrin, we used an electrophoretic mobility shift assay (EMSA) and fluorescence polarization (FP) analysis. For the EMSA, GreenB1 or RND at 17 nM concentration was incubated with α3β1-integrin at increasing concentrations and separated by electrophoresis on 3% agarose gel. Whereas the GreenB1 band decreased in intensity with increasing α3β1-integrin concentration (Figure 4A), no change was seen for RND (Figure 4B). The calculated K_D_ for GreenB/α3β1-integrin interaction was 15 nM (95% confidence interval [CI] 8–26 nM) (Figure 4C).

GreenB1 binding to CD44 and β1-integrin alone was further tested using FP. FP analysis revealed no binding of GreenB1 to the CD44 protein (Figure S5). Varying concentrations of β1-integrin were incubated with 10 nM of FAM-labeled GreenB1 or FAM-labeled RND. An increase in FP was observed for GreenB1 but not for RND (Figure 4D). A K_D_ value of 7 nM (95% CI 0–17 nM) was calculated using Prism 9.3.1 (GraphPad). The results from the EMSA and FP show that the GreenB1 aptamer binds to β1-integrin in the low nanomolar range.

### GreenB1 binding does not affect the amount of β1-integrin available for binding

**Figure 5:**
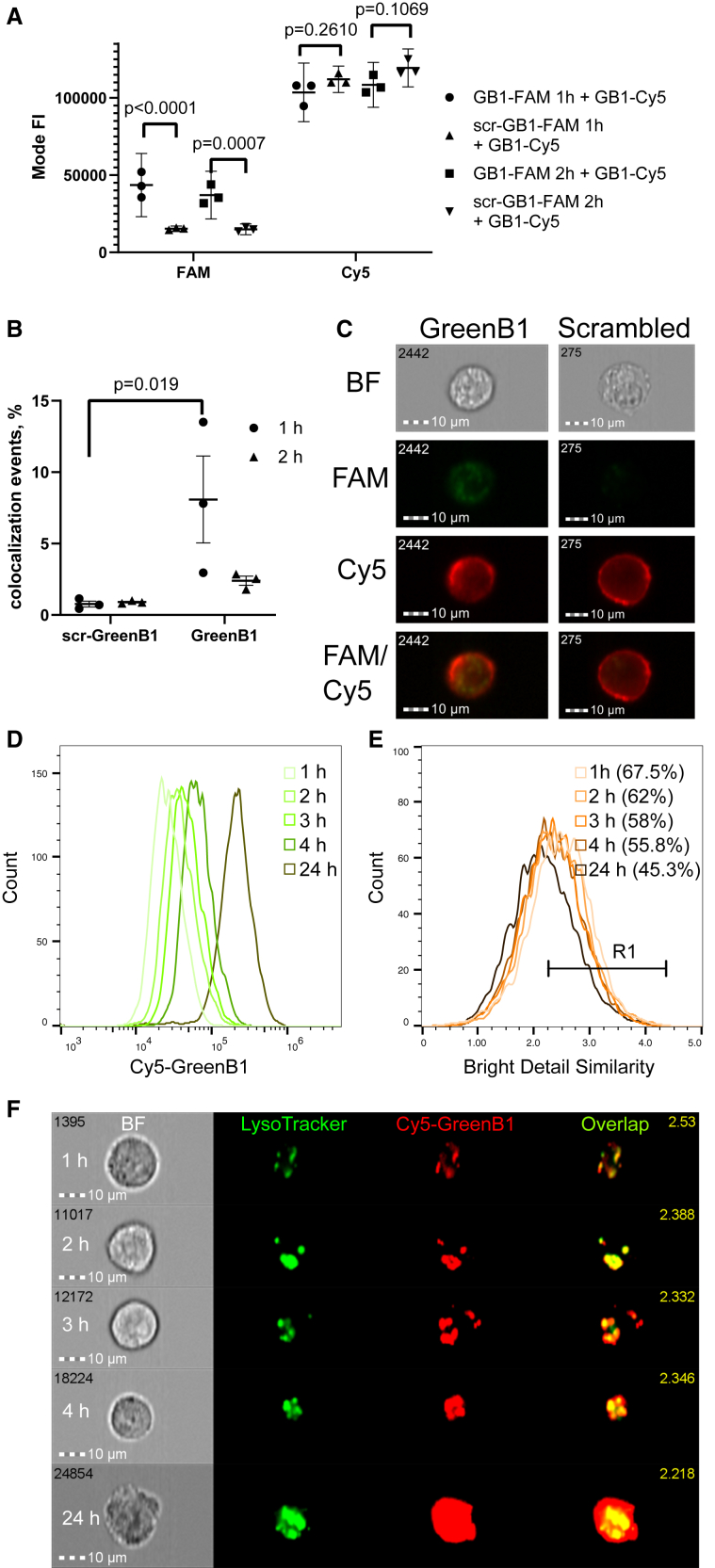
Cellular internalization cycle of the GreenB1 aptamer (A) FAM-labeled scr-GreenB1 aptamer library pre-incubation with MDA-MB-231 cells on a 6-well plate at different concentrations for 1 and 2 h, followed by incubation with 100 nM Cy5-GreenB1 at 4°C for 1 h. FAM-GreenB1 binding is statistically significantly higher compared with FAM-scr-GreenB1 (p < 0.0001 after 1 h and p = 0.0007 after 2 h pre-incubation), but Cy5-GreenB1 binding afterward is not affected (p = 0.2610 after 1 h and 0.1069 after 2 h pre-incubation). Error bars indicate SD. (B) FAM-GreenB1 pre-incubation followed by Cy5-GreenB1 incubation resulted in statistically significantly more cell CL events than with FAM-scr-GreenB1 pre-incubation after 1 h (p = 0.019), but the difference was not statistically significant after 2 h. Error bars indicate SD. (C) Representative imaging flow cytometry images used for CL analysis. The number in the top left corner of each image is automatically assigned to each cell. (D) GreenB1 is stable in 10% FBS at 37°C for at least 6 h (Figure S6). (E) Cy5-labeled GreenB1 aptamer pulse-chase incubation (100 nM) with MDA-MB-231 cells in 6-well plates results in a time-dependent Cy5 fluorescence intensity increase. (F) Cy5-GreenB1 CL with LysoTracker Green shows the highest CL based on bright detail similarity between LysoTracker Green and Cy5-GreenB1 1 h after incubation and a slight decrease after 24 h. (G) Representative images of Cy5-GreenB1 CL with lysosomes at different time points.

To find out whether GreenB1 binding to β1-integrin has an impact on β1-integrin density on the cell surface, we tested GreenB1 binding dynamics by pre-incubating either FAM-scr-GreenB1 or FAM-GreenB1 with MDA-MB-231 cells at 200 nM for 1 or 2 h. After pre-incubation, FAM-scr-GreenB1/FAM-GreenB1-containing medium was removed, cells were collected, and both samples were incubated with 100 nM Cy5-GreenB1 on ice for 1 h. FAM-GreenB1 showed statistically significantly higher binding to cells compared with FAM-scr-GreenB1 at both time points. However, Cy5-GreenB1 binding after pre-incubation with either FAM-scr-GreenB1or FAM-GreenB1 was not statistically significantly different (Figure 5A). The lack of change indicated that the target protein remained available for aptamer binding irrespective of whether FAM-GreenB1 or FAM-scr-GreenB1 was used for pre-incubation. Moreover, FAM-scr-GreenB1 and Cy5-GreenB1 co-localization analysis showed that less than 1% of cells analyzed using imaging flow cytometry could be considered co-localization events. Cells pre-incubated with FAM-GreenB1 resulted in a statistically significantly higher fraction of co-localization events (8%) with Cy5-GreenB1 after 1 h (Figure 5B). The difference was not statistically significant after 2 h pre-incubation (2.4%) (Figure 5C). Cell pre-incubation with FAM-GreenB1 did not affect anti-β1-integrin antibody binding and did not show any co-localization between β1-integrin and GreenB1 (Figure S7). The incubation time points were chosen based on GreenB1 stability in the presence of 10% fetal bovine serum (FBS) for at least 6 h (Figure 5D).

### GreenB1 rapidly internalizes in cells and shows co-localization with LysoTracker-labeled vesicles

Aptamer uptake was further studied using pulse-chase fluorescence imaging. Cy5-labeled GreenB1 was incubated with MDA-MB-231 cells in complete culture medium at 100 nM for 1 h. The cells were collected immediately, or the aptamer-containing medium was replaced with fresh culture medium without the aptamer, and cells were incubated for an additional 2, 3, 4, and 24 h. 75 nM LysoTracker Green DND-26 was added to each sample before imaging flow cytometry. Co-localization analysis was done using IDEAS software based on bright detail similarity in both fluorescence channels. The intensity of the Cy5-GreenB1 signal increased over time, indicating that the uptake of GreenB1 into endocytic vesicles was fast and that the degradation and dissociation of the Cy5 fluorophore within the cells were slower (Figure 5E). The bright detail similarity was highest after 1 h (67.5% of cells were determined to be co-localization events) and decreased with each subsequent time point (2 h = 62%, 3 h = 58%, 4 h = 55.8%, 24 h = 45.3%), indicating release of Cy5 after degradation of GreenB1 (Figures 5F and 5G).

### GreenB1 co-localizes with β1-integrin on the cell surface

**Figure 6:**
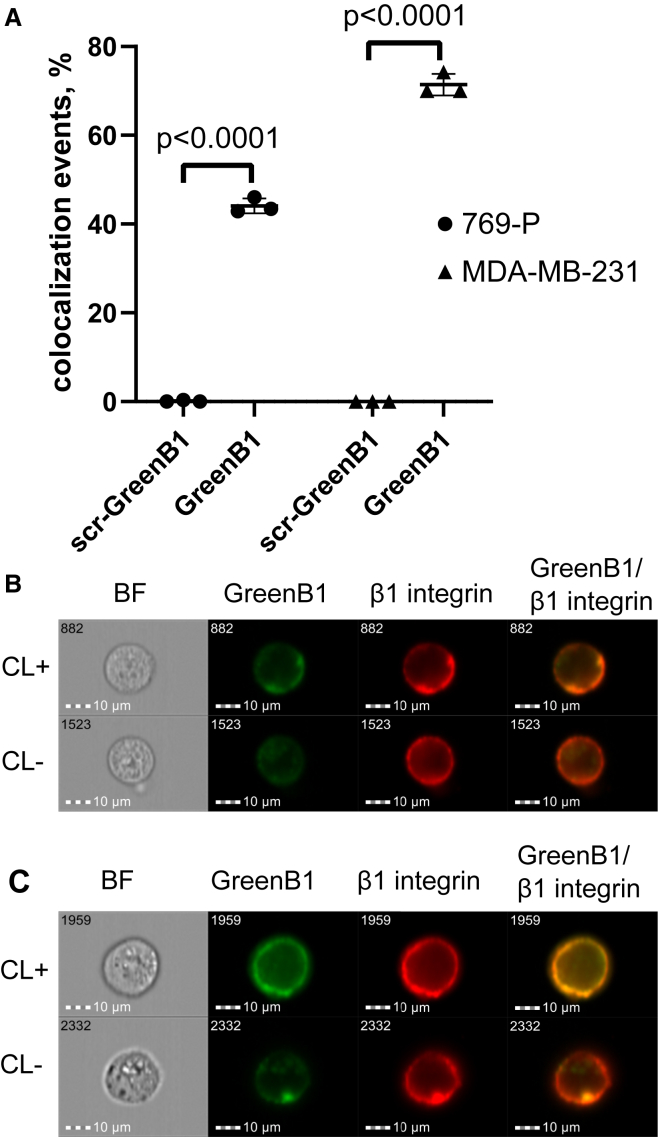
GreenB1 and β1-integrin CL analysis (A) FAM-scr-GreenB1 or FAM-GreenB1 CL with anti-β1-integrin-PE-Cy5 antibody on MDA-MB-231 and 769-P cells. Less than 1% of 769-P and MDA-MB-231 cells can be considered as having β1-integrin and scr-GreenB1 CL events compared with more than 40% of 769-P cells (p < 0.0001) and more than 70% of MDA-MB-231 (p < 0.0001) CL events between scr-GreenB1 and β1-integrin. Error bars indicate SD. (B and C) Representative images of 769-P (B) and MDA-MB-231 (C) cells considered as having CL events (CL+) or cells that are not considered as having CL events (CL−) based on bright detail similarity.

We next tested the ability of GreenB1 to co-localize with β1-integrin when incubated simultaneously with an anti-β1-integrin antibody to confirm β1-integrin as the target protein of the GreenB1 aptamer. Imaging flow cytometry bright detail similarity analysis showed that less than 1% of 769-P and MDA-MB-231 cells had co-localization events between scr-GreenB1 and β1-integrin. However, more than 40% of 769-P and more than 70% of MDA-MB-231 cells were classified as having co-localization events between GreenB1 and β1-integrin (Figure 6A). GreenB1 co-localization events (CL+) in 769-P and MDA-MB-231 cell lines (Figures 6B, CL+, and 6C, CL+) mainly showed membrane staining. Cells that were classified as not having co-localization events (CL−) (Figures 6B, CL−, and Figure 6C, CL−) had β1-integrin staining limited to the cell surface, while GreenB1 staining was also observable as specks closer to the center of cells.

### β1-Integrin silencing results in reduced GreenB1 binding

**Figure 7:**
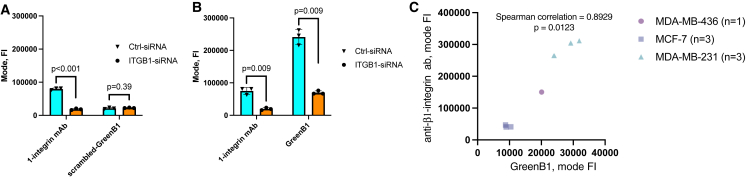
β1-Integrin expression changes correlate with GreenB1 binding (A) Anti-β1-integrin antibody binding is significantly reduced when β1-integrin is silenced (p < 0.001), but the control scr-GreenB1 aptamer’s binding to MDA-MB-231 cells is unaffected (p = 0.39). Error bars indicate SD. (B) The amount of GreenB1 bound to MDA-MB-231 cells (p = 0.009) and anti-β1-integrin antibody binding (p = 0.009) is decreased by knockdown of β1-integrin expression. Error bars indicate SD. (C) Fluorescence intensity from GreenB1 binding correlates with fluorescence intensity from β1-integrin expression levels tested by antibody binding (Spearman correlation = 0.8929, p = 0.0123) on the MDA-MB-231, MCF-7, and MDA-MB-436 cell lines.

We used small interfering RNA (siRNA) to reduce β1-integrin expression in MDA-MB-231 cells and further validate β1-integrin as the target protein for GreenB1. Compared with control siRNA, ITGB1 siRNA proved to statistically significantly reduce the expression of β1-integrin in MDA-MB-231 cells (Figures 7 and S8). Scr-GreenB1 aptamer binding to MDA-MB-231 cells was not statistically significantly different (p = 0.39) when comparing the amount of scr-GreenB1 bound to cells transfected with control siRNA or ITGB siRNA (Figure 7A). However, when β1-integrin was knocked down, GreenB1 binding to MDA-MB-231 cells was statistically significantly reduced (p = 0.009) and by a similar fraction as β1-integrin antibody binding (Figure 7B).

### GreenB1 binding correlates with β1-integrin expression level

We compared GreenB1 binding and anti-β1-integrin antibody binding to the MDA-MB-231, MDA-MB-436, and MCF-7 cell lines to see whether β1-integrin expression levels detected by antibody are in alignment with GreenB1 binding. GreenB1 and anti-β1-integrin antibody binding shows a statistically significant (p = 0.0123) positive correlation (Spearman r = 0.8929) (Figures 7C and S9). Correlation was not statistically significant (p = 0.0881) between the scr-GreenB1 and isotype control antibody (Figure S10).

## Discussion

TNBC is the most lethal of the breast cancer subtypes, with an estimated median overall survival time for metastatic TNBC of 10–13 months. However, when detected early, at stage I, TNBC has a 5-year survival rate of 85%, which is lower than for other breast cancer subtypes.^46^ Chemotherapy in combination with ICIs has been demonstrated to improve median progression-free survival and median overall survival in PD-L1-positive subgroups,^13^^,^^47^ but an ideal therapeutic approach remains elusive.^48^ Precision-guided interventions hold promise for delivering therapeutic agents to tumors, and aptamers with high selectivity toward target molecules are promising candidates for targeted therapy or diagnostics purposes. We demonstrate that, at low nanomolar concentrations, aptamer GreenB1 selectively binds to the TNBC cell lines MDA-MB-231 and MDA-MB-436 but not to the ER- and PR-expressing breast cancer cell line MCF-7. On the cell surface, GreenB1 interacts with β1-integrins. GreenB1 is subsequently internalized via the endolysosomal uptake pathway, while β1-integrin is recycled back to the cell surface.

GreenB1-based targeting strategies can likely be used for precision delivery of drugs and imaging agents to β1-integrin-positive solid tumors other than TNBC. Integrins have been used extensively in cancer therapy affinity targeting efforts. Several antibody-based integrin αV- and αVβ3/β1/β5-targeting therapies have been tested in phase I/II clinical trials with disappointing results.^48^ Antibody delivery to poorly vascularized tumor tissue could be hampered by their large molecular weight. The smaller iRGD peptide targeting αVβ3/β5 integrins has shown promising preclinical results for pancreatic ductal adenocarcinoma therapy^49^ and is now being investigated in a phase II clinical trial (ClinicalTrials.gov: NCT03517176). The NRP-1 binding CendR motif in iRGD promotes extravasation into the tumor.^50^ GreenB1 is suited for development into an affinity-targeting ligand that is several orders of magnitude smaller than antibodies. However, detailed *in vivo* studies are required to support our *in vitro* findings.

GreenB1 CL with acidic vesicles suggests that it is internalized in cells and likely being trafficked via the route established previously for oligonucleotide delivery. According to it, oligonucleotides are transported to lysosomes for degradation.^51^ FAM fluorescence is reduced at an acidic pH, resulting in relatively low FAM-GreenB1 signal intensity observed during the pre-incubation study, compared with the binding data at different concentrations, further suggesting that GreenB1 is internalized within acidic vesicles.^52^

Lysosome-targeting chimeras (LYTACs) take advantage of lysosome shuttling proteins to target membrane-bound and extracellular proteins for degradation and could be used to act on currently “undruggable” proteins.^53^ A bispecific aptamer-based LYTAC system has used insulin growth factor type II receptor (IGF-IIR) as a lysosome shuttling component to degrade targeted proteins.^54^ GreenB1 trafficking to acidic vesicles implies that more research on the application of this aptamer to produce LYTACs that work via the β1-integrin re-cycling route is warranted. It has been shown recently that integrin-facilitated protein degradation can occur through integrin recycling using RGD peptides.^55^ Furthermore, it is likely that, by modifying GreenB1-based targeting to allow endolysosomal escape, the system can be adapted for delivery of payloads into the cytosol and other intracellular compartments. This will be particularly important for large siRNA and peptide cargoes with a polar and charged character that are unable to translocate efficiently into the cytosol to perform their biological activity.^51^^,^^56^

Unmodified aptamers have a circulation half-life of minutes to hours and are degraded in serum by exonucleases. GreenB1 has been shown to stay intact for 6 h and to be partially degraded after 24 h and completely degraded after 48 h. The circulating half-life of GreenB1 can be modified by adding high-molecular-weight compounds, such as polyethylene glycol (PEG), creating multivalent constructs larger than the glomerular filtration rate cutoff (50–60 kDa),^16^^,^^57^ or circularizing the aptamer to make it less susceptible to nuclease digestion.^22^ GreenB1 can be linked to cytotoxic chemicals via a lysosome-sensitive linker^58^ or liposomes containing an anticancer payload^59^ to assess its ability to diminish tumor burden.

In addition to applications in targeted delivery, GreenB1 may have inherent functional activity because of modulating the status of its target integrins. Integrins are known to profoundly regulate cell migration, survival, and proliferation. Compared with many cell-surface proteins that are degraded or do not change their location after ligand binding, integrins are constantly trafficked and recycled within cells.^60^ Integrin expression modulation is linked to cancer invasion, formation of metastatic lesions, tumor growth, and development of resistance to treatment.^61^ In breast cancer, the receptor tyrosine kinase c-Met can replace α5-integrin as a β1-integrin binding partner, forming a complex that drives cancer cell migration because of higher affinity to fibronectin.^62^ In TNBC, blocking the β1-integrin and Talin-1 (TLN1) interaction has been described recently as a potential therapeutic target.^63^ Silencing of β1-integrin has been shown to increase the sensitivity to cancer drugs and inhibit cancer cell migration and invasion.^64^ β1-Integrin silencing has also been proposed as a promising therapeutic approach for reducing radioresistance in non-small cell lung cancer.^65^ β1-Integrin is a required protein for forming vasculogenic mimicry, a tumor blood supply mechanism where cancer cells form blood vessel-like structures.^66^ GreenB1 has a high affinity for β1-integrin, suggesting that it could be used therapeutically to disrupt β1-integrin interactions with TLN1 or c-Met, altering TNBC cell invasiveness. Alternatively, research into a GreenB1-based strategy that silences β1-integrin activities in TNBC and thus increases susceptibility to existing therapies is necessary.

As a technical advancement, we adopted a proximity labeling-based approach (widely used to study protein-RNA/DNA and protein-protein interactions) to identify GreenB1 engagement partners. Compared with extract-based techniques, such as affinity precipitation, proximity ligation has the advantage of yielding less background and more relevant hits. To generate reactive species from a substrate, biotin ligases or peroxidases connected to a targeting moiety, such as an aptamer, are used. The activated substrate then covalently bonds to neighboring proteins and can be utilized to pull down proteins close to the binding point.^67^ Our unpublished studies show that a similar approach can be used to identify binding partners for other targeting ligands, such as peptides, and that the technique can be even applied to *in vivo* interaction studies (M.H., unpublished data).

In summary, here we report a new β1-integrin-binding aptamer, GreenB1, that selectively binds TNBC cells *in vitro* and is quickly internalized in cells but does not affect the amount of β1-integrin available for binding on the cell surface. GreenB1 translational applications are of great interest in the future and might lead to innovative targeted protein breakdown or therapeutic approaches.

## Materials and methods

### *In vitro* cell culture

The ER- and PR-positive breast adenocarcinoma MCF-7 cell line (HTB-22, ATCC) was cultivated in Dulbecco’s modified Eagle’s medium (DMEM; D6429, Sigma-Aldrich) supplemented with 10% FBS (F7524, Sigma-Aldrich) and 0.01 mg/mL human recombinant insulin. The TNBC cell lines MDA-MB-231 (HTB-26, ATCC) and MDA-MB-436 (HTB-130, ATCC) were cultivated in DMEM with 10% addition of FBS. The renal cell carcinoma cell line 769-P (CRL-1933, ATCC) was expanded in Roswell Park Memorial Institute 1640 (RPMI 1640) medium (61870-010, Gibco) with 10% FBS added. All culture media were supplemented with 100 U/mL penicillin/streptomycin (15140-122, Thermo Fisher Scientific) and kept at 37°C in a 95% humidified and 5% CO_2_ atmosphere.

### Aptamers and buffers

A FAM or biotin-labeled or unlabeled single-stranded DNA (ssDNA) RND containing constant primer binding regions and a 40-nt randomized region (5′-FAM/biotin-ATCCAGAGTGACGCAGCANNNNNNNNNNNNNNNNNNNNNNNNNNNNNNNNNNNNNNNNTGGACACGGTGGCTTAGT-3′), FAM labeled scr-GreenB1 (5′-FAM-ATCCAGAGTGACGCAGCAGGTGGAAGGGGTAACTACGTGGGGAGGTGGTAGGGGTGGGTGGACACGGTGGCTTAGT-3′), and a FAM-, Cy5-, or biotin-labeled or unlabeled ssDNA aptamer GreenB1-containing primer binding constant regions and 40-nt sequence in between (5′-FAM/Cy5/biotin-ATCCAGAGTGACGCAGCATGGGGGTAGTGGTGGTTAGGAGTGGAGGCGAGGAGAGCGGTGGACACGGTGGCTTAGT-3′) were purchased from Integrated DNA Technologies. Oligonucleotides were diluted to 100 μM concentration using DNase and RNase-free water. Aptamers were folded at 10 μM or 1 μM concentration in folding or binding buffer at 95°C for 5 min and then cooled down to room temperature (RT) for at least 15 min. The binding buffer contained 5 mM MgCl_2_, 4.5 mg/mL D-glucose, 0.1 mg/mL baker’s yeast tRNA (for experiments using RND as a control), or salmon sperm DNA (experiments using scr-GreenB1 as a control) (15632011, Thermo Fisher Scientific) and 1 mg/mL bovine serum albumin (BSA; A9647, Sigma-Aldrich) in MgCl_2_ and CaCl_2_-free phosphate-buffered saline (PBS; D8537, Sigma-Aldrich, containing K^+^ at 4.45 mM and Na^+^ at 157 mM concentration). The folding buffer contained 5 mM MgCl_2_ in PBS. NUPACK software was used to predict the secondary structure of GreenB1 and scr-GreenB1.^68^

### Aptamer binding to MCF-7, MDA-MB-231, and MDA-MB-436 cells

FAM-scr-GreenB1 and FAM-GreenB1 were folded at 1 μM in binding buffer and diluted (500, 250, 125, 25, and 5 nM). MCF-7, MDA-MB-231, and MDA-MB-436 cells were cultivated in a T75 flask (Sarstedt) until 80% confluence. Cells were washed with PBS and dissociated using non-enzymatic cell-dissociation buffer (25-056-CI, Corning) for 5–9 min, followed by addition of complete culture medium, centrifugation at 300 × *g* for 5 min and removal of the supernatant. Cells were washed twice with binding buffer, split into samples, and resuspended with different concentrations of FAM-scr-GreenB1 or FAM-GreenB1 (n = 3 for each concentration, aptamer and cell line). Samples were incubated on ice for 1 h, washed twice with washing buffer, resuspended in 40 μL of binding buffer, and analyzed using an Amnis ImageStream^X^ Mk II imaging flow cytometer and IDEAS software (Luminex) or an Accuri C6 Plus (BD Biosciences) flow cytometer. Statistical significance was determined using unpaired t tests, and statistical significance was adjusted for multiple comparisons using the Holm-Šídák method. Apparent K_D_ was calculated by subtracting the FAM-scr-GreenB1 non-specific signal from the FAM-GreenB1 signal, and data were fitted using one site-specific binding model. All calculations were done using Prism 9.3.1 (GraphPad).

### Surface β1-integrin availability after GreenB1 binding *in vitro*

FAM-scr-GreenB1 or FAM-GreenB1 aptamers were folded at 10 μM concentration in binding buffer and incubated at 200 nM concentration in complete growth medium supplemented with 10% FBS, with MDA-MB-231 cells grown on a 6-well plate at 37°C in an incubator for 1 or 2 h (n = 3 for each aptamer and each time point). Cells were removed from a 6-well plate using a cell scraper and incubated on ice with the Cy5-GreenB1 aptamer at 100 nM concentration or anti-β1-integrin-phycoerythrin (PE)-Cy5 antibody for 1 h. Cells were washed with binding buffer twice, resuspended in 30 μL of binding buffer, and analyzed using an Amnis ImageStream^X^ Mk II imaging flow cytometer and IDEAS software (Luminex). Statistical significance was determined using 2-way ANOVA, and statistical significance was adjusted for multiple comparisons using Šídák’s multiple-comparisons test. All calculations were done using Prism 9.3.1 (GraphPad).

### Pulse-chase incubation and lysosome co-localization

MDA-MB-231 cells were cultivated in a 6-well plate until reaching 80% confluence. The Cy5-GreenB1 aptamer was folded in folding buffer at 10 μM concentration and diluted in 1 mL complete growth medium supplemented with 10% FBS to 100 nM before adding to cells. Cells were incubated with Cy5-GreenB1 for 1 h, which was replaced with complete growth medium; afterward, cells were removed for further processing using a non-enzymatic cell dissociation reagent. Cells were analyzed 1, 2, 3, 4, and 24 h after adding Cy5-GreenB1. After dissociation, cells were washed twice with PBS/0.1% BSA, resuspended in 100 μL PBS/0.1% BSA, and kept on ice. Before imaging flow cytometry, cells were centrifuged at 300 × *g* for 5 min and resuspended in 30 μL of 75 nM LysoTracker Green (L7526, Thermo Fisher Scientific). Samples were analyzed using an Amnis ImageStream^X^ Mk II imaging flow cytometer (Luminex).

### Serum degradation study

GreenB1 was folded in PBS/5 mM MgCl_2_ at 1 μM concentration as described previously, 10% of FBS was added to the aptamer, and the mixture was incubated in a heat block at 37°C. Samples were taken after 1, 2, 3, 6, 24, and 48 h (n = 3) and kept at −20°C until further use. From each sample, 1 μL was mixed with loading dye and water and loaded on 3% agarose gel stained with SYBR Gold nucleic acid stain (S11494, Thermo Fisher Scientific). The gel was run at 100 V for 40 min.

### Proximity labeling of the GreenB1 target protein

The tyramide-Alexa Fluor 555 working solution was prepared by combining 50 μL of 20× reaction buffer ( omponent C3 from B40933), 1,000 μL purified water, 10 μL of tyramide-Alexa Fluor 555 reagent (component C1 from B40933), and 10 μL of 0.15% hydrogen peroxide. The tyramide-biotin working solution was prepared by combining 50 μL of 20× reaction buffer (component C3 from B40933), 1,000 μL purified water, and 10 μL of 0.15% hydrogen peroxide and adding Tyramide-biotin (LS-3500, Iris Biotech) at 500 μM final concentration.

GreenB1-biotin, unlabeled GreenB1, RND-biotin, and unlabeled RND oligonucleotides were diluted in 500 μL folding buffer to 1 μM concentration and folded as described in the previous section. HRP-conjugated streptavidin (component B, 500 μL) from the Alexa Fluor 555 Tyramide SuperBoost Kit (B40933, Thermo Fisher Scientific) was added to folded oligonucleotides and incubated at RT for 30 min to create an oligonucleotide-biotin-streptavidin-HRP complex. The mixture was transferred to a 100-kDa molecular weight cut-off (MWCO) Amicon Ultra-4 centrifugal filter unit (UFC810008, Merck), centrifuged at 7,500 × *g*, refilled four times to remove the unbound aptamer, and finally concentrated to approximately 100 μL. The resulting complex was diluted to 500 μL and added to cells for performing confocal microscopy or labeled protein pull-down using magnetic streptavidin beads (65001, Thermo Fisher Scientific) afterward.

For confocal microscopy, MDA-MB-231 cells were cultivated in an 8-well culture slide (354118, Falcon). Cells were washed twice with PBS before applying oligonucleotide-biotin-streptavidin-HRP complexes, followed by incubation at 37°C for 1 h. The medium was aspirated, and cells were washed 3 times with folding buffer before adding 100 μL of tyramide-Alexa Fluor 555 working solution to each well. The reaction was stopped after 2 min by adding 100 μL of 1× stop reagent (100 μL of component D in DMSO from B40933 and 1,100 μL of PBS) to each well. Cells were washed 3 times with PBS, fixed with 4% formaldehyde at RT for 10 min, and washed twice with PBS. Nuclei were stained with DAPI (D1306, Thermo Fisher Scientific) at RT for 5 min and washed once with PBS. Chambers were removed from the slide, mounting medium and coverslip were added to the slide, and it was imaged.

For the pull-down experiment, MDA-MB-231 cells were cultivated in a T75 flask, washed with PBS once, and dissociated using non-enzymatic cell-dissociation buffer (CellStripper, Corning) for 5–9 min, followed by addition of complete culture medium. The cell suspension was split into the necessary number of samples (approximately 1 × 10^6^ cells per sample) and centrifugated for 5 min at 300 × *g*, and the supernatant was removed. Cells were resuspended in 500 μL of folding buffer and 500 μL of oligonucleotide-biotin-streptavidin-HRP complexes, followed by incubation at RT for 1 h in an end-over-end rotator. Cells were then centrifuged at 300 × *g*, washed twice with PBS, and resuspended in 100 μL of tyramide-biotin working solution. The reaction was stopped after 2 min by adding 100 μL of 1× stop reagent (100 μL of component D in DMSO from B40933 and 1,100 μL of PBS) to each sample. Samples were washed with PBS and centrifuged at 300 × *g* for 5 min. The samples were either subjected to flow cytometry to confirm biotinylation or lysed for pull-down of biotinylated proteins. For flow cytometry, samples were incubated with Streptavidin-DyLight-488 (21832, Thermo Fisher Scientific) at 20 μg/mL in folding buffer for 10 min, washed 3 times with PBS, fixed with 4% formaldehyde, washed 3 times with PBS, resuspended in 200 μL PBS/0.1% BSA, and analyzed using flow cytometry (BD Accuri C6 Plus). For protein pull-down, samples were lysed by adding 200 μL of sample lysis buffer (50 μL of 4× sample buffer, 20 μL of 10% of n-dodecyl-β-D-maltoside [DDM] from the NativePage Sample Prep Kit [BN2008, Thermo Fisher Scientific], and 130 μL of PBS) and pipetting to solubilize the proteins. Lysed samples were centrifuged at greater than 20,000 × *g* at 4°C for 30 min. The supernatant was collected and added to 20 μL of Dynabeads MyOne Streptavidin C1 beads (65001, Thermo Fisher Scientific) per sample. The lysate was incubated with magnetic beads at RT on an end-to-end rotator for 30 min. Beads were washed four times with 200 μL of sample lysis buffer. Beads were transferred to a new 1.5-mL centrifuge tube after each wash. Elution was achieved by adding 30 μL of 25 mM biotin in lysis buffer and heating at 95°C for 5 min. The biotin elution strategy was adapted from Cheah and Yamada.^69^ Elution was repeated two times, and the supernatant was collected. The third elution step was done by adding 30 μL of reducing sample buffer and heating at 95°C for 5 min.

### Tris/glycine gel electrophoresis and MS

Reducing sample loading buffer (2 μL) was added to 10 μL of each elution from streptavidin beads after proximity labeling. Samples were heated at 95°C for 5 min and loaded on 12% Mini-PROTEAN TGX precast protein gel (4561043, Bio-Rad). The gel was run using 1× Tris/glycine running buffer at 100 V for 90 min. The gel was stained using the SilverQuest Silver Staining Kit (LC6070, Thermo Fisher Scientific). The bands of interest were cut out and sent for MS proteomics analysis at the University of Tartu Proteomics core facility (https://www.tuit.ut.ee/en/research/proteomics-core-facility). Figure 3D was prepared using R^70^ in RStudio (v.2021.9.2.382)^71^ and the packages readxl,^72^ ggplot2,^73^ and ggrepel.^74^

### EMSA with α3β1-integrin

GreenB1 or RND aptamers were folded at 1 μM concentration and diluted to 35 nM using a folding buffer with 5% glycerol. The α3β1-integrin protein complex (2840-A3-050, R&D Systems, 20 μL) at 360 nM (100 μg/mL) in PBS was diluted to 180 nM (molar concentrations were calculated based on SDS-PAGE migration of each protein under reducing conditions, 150 kDa for α3-integrin and 125 kDa for β1-integrin) using a folding buffer with 10% glycerol. Further dilutions were prepared using a folding buffer with 5% glycerol. To each 10 μL of α3β1-integrin dilutions, 10 μL of 35 nM of GreenB1 or RND was added. Final concentration for aptamers was 17.5 nM, and α3β1-integrin concentrations were 90, 45, 22.5, 11.25, 5.61, 2.8, 1.4, and 0.7 nM. The mixture was then incubated at RT for 2 h and loaded on 3% agarose gel prepared using 0.5× Tris/boric acid buffer without ethylenediaminetetraacetate (EDTA) and run at 180 V in a cold room (4°C) using 0.5× Tris/boric acid as running buffer for 30 min. The gel was stained with SYBR Gold nucleic acid stain in 0.5× Tris/boric acid buffer for 30 min and destained in purified water for 10 min. K_D_ was calculated from triplicate measurements using Prism 9.3.1 (GraphPad) using one site-specific binding equation (Y = Bmax × X/[K_D_ + X]).

### FP assay with β1-integrin and CD44

FP reactions (25 μL) were set up in black, non-transparent, flat-bottom, 384-well microplates (3821, Corning). Each reaction contained PBS supplemented with 5 mM MgCl_2_, 0.05% Tween 20, 10 nM FAM-labeled GreenB1 aptamer or FAM-labeled RND, and varying concentrations of β1-integrin (10587-H08H1, SinoBiological), CD44v5 (11087-CD, R&D Systems), or CD44v6 (11237-CD, R&D Systems). Reactions were performed in triplicate. Measurements were recorded on a Hidex Sense microplate reader equipped with 485-nm/535-nm optical filters using 100 flashes, medium lamp power, and a PMT voltage of 750 V. Titration data were fitted in Prism 9.3.1 (GraphPad) using the following equation:

LR=((X+Ltot+KD)−SQRT((X+Ltot+KD)ˆ2–4∗X∗Ltot))/2

Y=BKG+FR∗LR

where X is the concentration of integrin-β1/CD44v5/CD44v6 (serial 2-fold dilutions); Ltot is the total concentration of the aptamer (fixed); PR is the fluorescence ratio, a unitless constant (fitted); BKG is the background polarization of the unbound aptamer (fitted); K_D_ is the dissociation constant (fitted); and Y is the fluorescence polarization (recorded).

### GreenB1 CL with an anti-β1-integrin antibody on 769-P and MDA-MB-231 cells

The MDA-MB-231 and 769-P cell lines were cultured in T75 flasks until greater than 80% confluence. Cells were washed with PBS twice and dissociated with a non-enzymatic cell dissociation solution for 7–9 min in a cell culture incubator. After dissociation, cells were washed with PBS/0.1% BSA and split into the necessary number of samples. The negative control sample was incubated with 100 μL PBS/0.1% BSA, the isotype control sample was resuspended with 100 μL PBS/0.1% BSA and 20 μL of PE-Cy5 mouse immunoglobulin G1 (IgG1) isotype control (555750, BD Biosciences), scrambled aptamer control samples (n = 3) were incubated with FAM-scr-GreenB1 at 100 nM final concentration and 20 μL PE-Cy5 mouse anti-human CD29 antibody (559882, BD Biosciences), and target samples (n = 3) were incubated with FAM-GreenB1 at 100 nM final concentration and 20 μL PE-Cy5 mouse anti-human CD29 antibody. Incubation was performed on ice for 1 h. After incubation, samples were washed twice with PBS/0.1% BSA, resuspended in 30 μL PBS/0.1% BSA, and subjected to imaging flow cytometry. The compensation matrix was prepared using separate single-stained samples labeled with either FAM-GreenB1 or PE-Cy5 mouse anti-human CD29 antibody. Samples were analyzed using an Amnis ImageStream^X^ Mk II imaging flow cytometer (Luminex). Statistical significance was determined using 2-way ANOVA, and statistical significance was adjusted for multiple comparisons using Šídák’s multiple-comparisons test. All calculations were done using Prism 9.3.1 (GraphPad).

### β1-Integrin silencing using siRNA

MDA-MB-231 cells were cultured in 6-well plates until greater than 80% confluence. Control siRNA (4390843, Thermo Fisher Scientific) or ITGB1 siRNA (s7574, Thermo Fisher Scientific) was mixed with MIRFECT (RNAexact) in Opti-MEM medium I reduced serum medium (31985062, Thermo Fisher Scientific) incubated at RT for 30 min and added to cells at a final siRNA concentration of 20 nM. Cell culture medium was changed to fresh cell culture medium without siRNA after 6 h. Cells were washed with PBS twice and dissociated with a non-enzymatic cell dissociation solution for 7–9 min 48 h post transfection. Dissociated cells were incubated on ice with either 100 μL FAM-scr-GreenB1 aptamer (n = 3) or FAM-GreenB1 (n = 3) at 200 nM concentration and 10 μL PE-Cy5 mouse anti-human CD29 antibody (559882, BD Biosciences). After incubation, cells were washed twice with binding buffer and analyzed using an Accuri C6 Plus (BD Biosciences) flow cytometer. Unpaired t test with Welch correction, adjusted for multiple comparisons using the Holm-Šídák method, was carried out using Prism 9.3.1 (GraphPad).

### GreenB1 and anti-β1-integrin antibody binding level correlation

FAM-GreenB1 and FAM-scr-GreenB1 were diluted with binding buffer to 1 μM concentration and folded. MDA-MB-231, MCF-7, and MDA-MB-436 cells were grown in T75 flasks until greater than 80% confluence, washed with PBS twice, and dissociated with non-enzymatic cell dissociation buffer for 7–9 min. FAM-GreenB1 was added at a final concentration of 200 nM along with 20 μL of PE-Cy5 mouse anti-human CD29 antibody (559882, BD Biosciences). FAM-scr-GreenB1 was added at a final concentration of 200 nM along with 20 μL of PE-Cy5 mouse IgG1 isotype control (555750, BD Biosciences). Samples were incubated on ice for 1 h, washed twice with binding buffer, and analyzed using an Amnis FlowSight imaging flow cytometer. Spearman correlation between FAM-GreenB1 and PE-Cy5 mouse anti-human CD29 antibody fluorescence intensities was calculated using Prism 9.3.1 (GraphPad).

## Data and code availability

Fluorescence and confocal microscopy images (.oib format under “GreenB1-confocal-images”), imaging flow cytometry data (.rif, .cif, .daf, and .cpm files) from GreenB1 concentration-dependent binding to MCF-7 and MDA-MB-231 cells, pre-incubation experiment with MDA-MB-231 cells, pulse-chase experiments with MDA-MB-231 cells, GreenB1 and β1-integrin CL study with MDA-MB-231 cells, MS proteomics (.raw and .xlsx data table under “greenb1-ms-proteomics”), and flow cytometry data from the siRNA experiment and MDA-MB-436 binding experiment have been deposited in BioStudies^75^ and are available at https://www.ebi.ac.uk/biostudies/studies/S-BSST857.

The MS proteomics data have been deposited in the ProteomeXchange Consortium^76^ via the PRIDE^77^ partner repository with the dataset identifiers PXD034982.

## Supplemental information

Document S1. Figures S1–S10 and Table S1

Document S2. Article plus supplemental information
