# Supplementary material for: Targeting triple-negative breast cancer cells with a β1-integrin binding aptamer
Source: Mol Ther Nucleic Acids. 2023 Aug 16;33:871–84. doi: 10.1016/j.omtn.2023.08.015 (PMC10481362; doi:10.1016/j.omtn.2023.08.015)
Supplement: Document S1. Figures S1–S10 and Table S1 [file mmc1.pdf]

OMTN, Volume *33*

Supplemental information

Targeting triple-negative breast cancer cells with a β1-integrin binding aptamer

Karlis Pleiko, Maarja Haugas, Vadims Parfejevs, Teodors Pantelejevs, Emilio Parisini, Tambet Teesalu, and Una Riekstina

Table S1. Proteins identified from proximity labelling using mass spectrometry.

Protein names	Gene names	GreenB1/RND	fraction of total GreenB1 intensity*1000
Integrin beta-1	ITGB1	766	22.80
Integrin alpha-3	ITGA3	761650000	4.08
CD44 antigen	CD44	215690000	1.16
Kinectin	KTN1	17	0.99
Actin, cytoplasmic 1	ACTB	15	0.77
Cell surface glycoprotein MUC18	MCAM	134760000	0.72
Isoform 4 of Sodium/potassium-transporting ATPase subunit alpha-1	ATP1A1	81395000	0.44
Isoform Alpha-6X2A of Integrin alpha-6	ITGA6	73210000	0.39
Histone H4	H4C1	56999000	0.31
Integrin alpha-5	ITGA5	46882000	0.25
Isoform 3 of Integrin alpha-V	ITGAV	43403000	0.23
Protein EVI2B	EVI2B	37945000	0.20
Keratin, type II cytoskeletal 4	KRT4	33353000	0.18
Isoform 2 of Keratin, type II cytoskeletal 80	KRT80	25	0.18
Immunoglobulin heavy constant gamma 1 (Fragment)	IGHG1	30679000	0.16
Activated leukocyte cell adhesion molecule	ALCAM	22385000	0.12
14-3-3 protein sigma	SFN	17335000	0.09
Annexin A1	ANXA1	16258000	0.09
Integrin alpha-2	ITGA2	14651000	0.08
Ectonucleotide pyrophosphatase/phosphodiesterase family member 1	ENPP1	11468000	0.06
2-phospho-D-glycerate hydro-lyase	ENO1	10733000	0.06
Pyruvate kinase (Fragment)	PKM	9883200	0.05
Immunoglobulin heavy constant alpha 1 (Fragment)	IGHA1	9545600	0.05
Serpin B4	SERPINB4	8388700	0.04
Cytoskeleton-associated protein 4	CKAP4	6733400	0.04
Catalase	CAT	6601700	0.04
Fructose-bisphosphate aldolase	ALDOA	6407500	0.03
Protein-glutamine gamma-glutamyltransferase K	TGM1	3788500	0.02

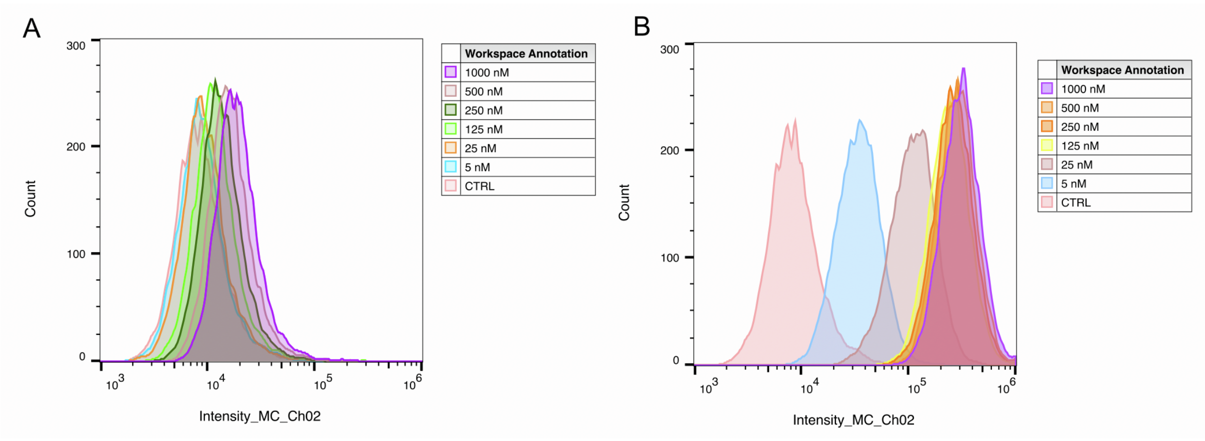

Figure S1. Flow cytometry analysis of GreenB1 binding to MDA-MB-231 at different concentrations. Representative chromatograms of FAM-scrambled-GreenB1 (A) and FAM-GreenB1 (B) binding to MDA-MB-231 at 0 nM to 1000 nM concentrations.

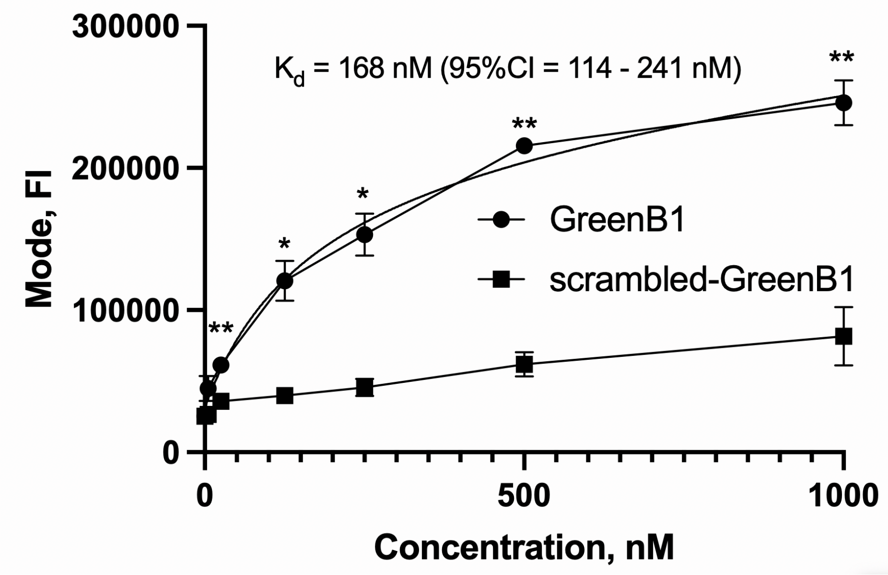

Figure S2. Flow cytometry analysis of GreenB1 binding to MDA-MB-436 at different concentrations. P-value corrected for multiple comparisons using Holm-Šídák method. Error bars indicate SD. *(p<0.05), **(p<0.01), ***(p<0.001).

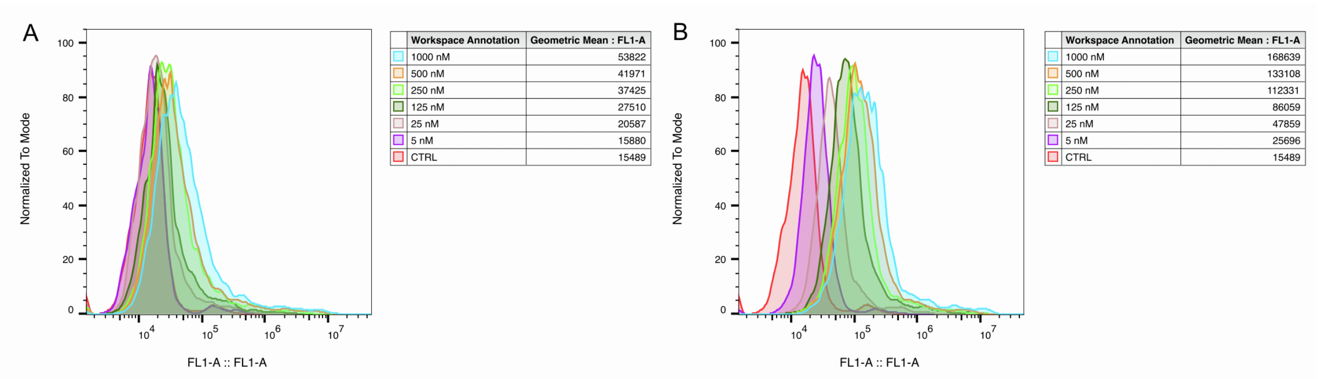

Figure S3. Flow cytometry analysis of GreenB1 binding to MDA-MB-436 at different concentrations. Representative chromatograms of FAM-scrambled-GreenB1 (A) and FAM-GreenB1 (B) binding to MDA-MB-436 at 0 nM to 1000 nM concentrations.

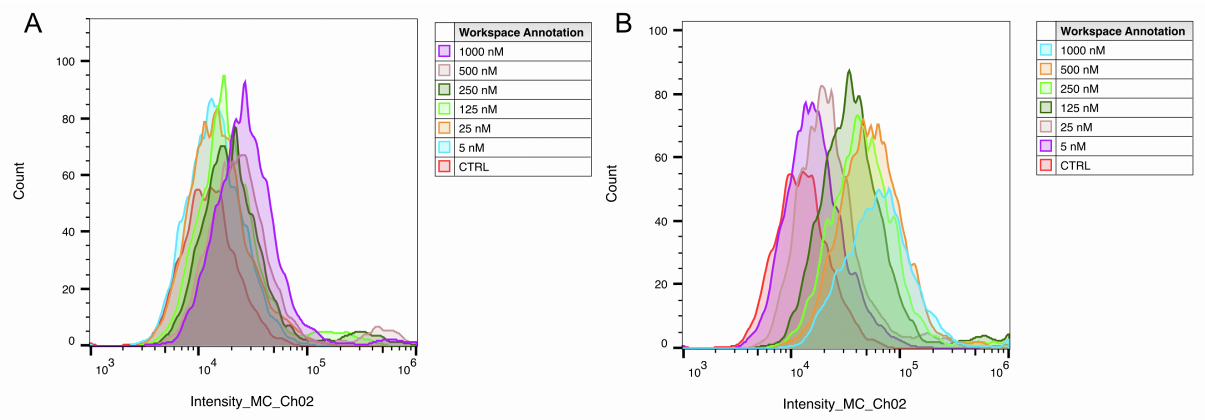

Figure S4. Flow cytometry analysis of GreenB1 binding to MCF-7 at different concentrations. Representative chromatograms of FAM-scrambled-GreenB1 (A) and FAM-GreenB1 (B) binding to MCF-7 at 0 nM to 1000 nM concentrations.

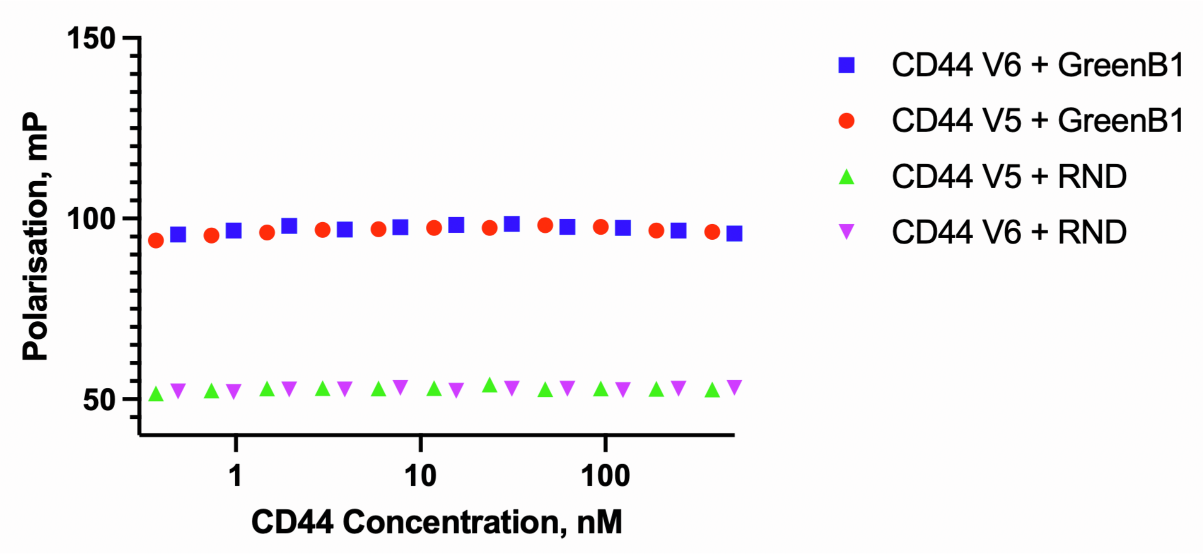

Figure S5. Fluorescence polarization using 10 nM of FAM-labelled GreenB1 aptamer or FAM-labelled RND and varying concentrations of CD44v5 and CD44v6 proteins.

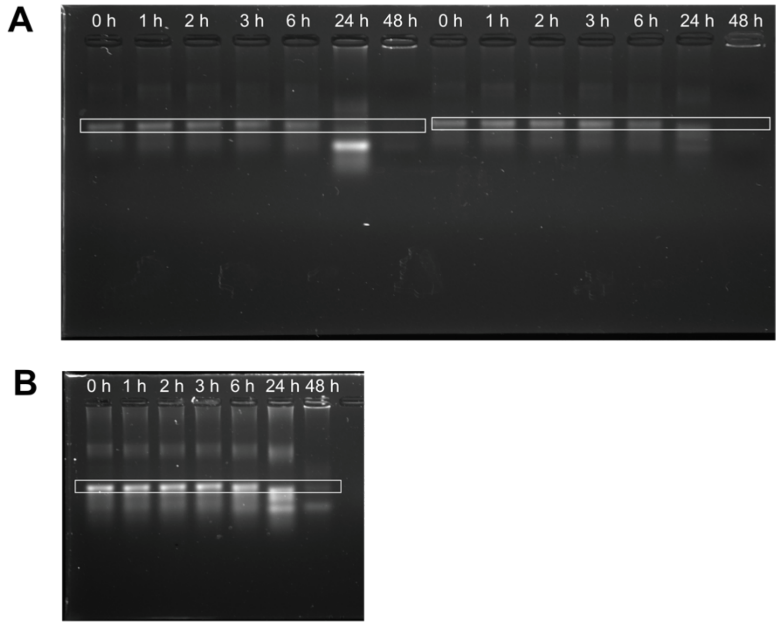

Figure S6. GreenB1 stability in 10% FBS. Two replicates ran on one gel (A) and one replicate ran on a separate gel (B) show partial degradation after 24 h and complete degradation after 48 h.

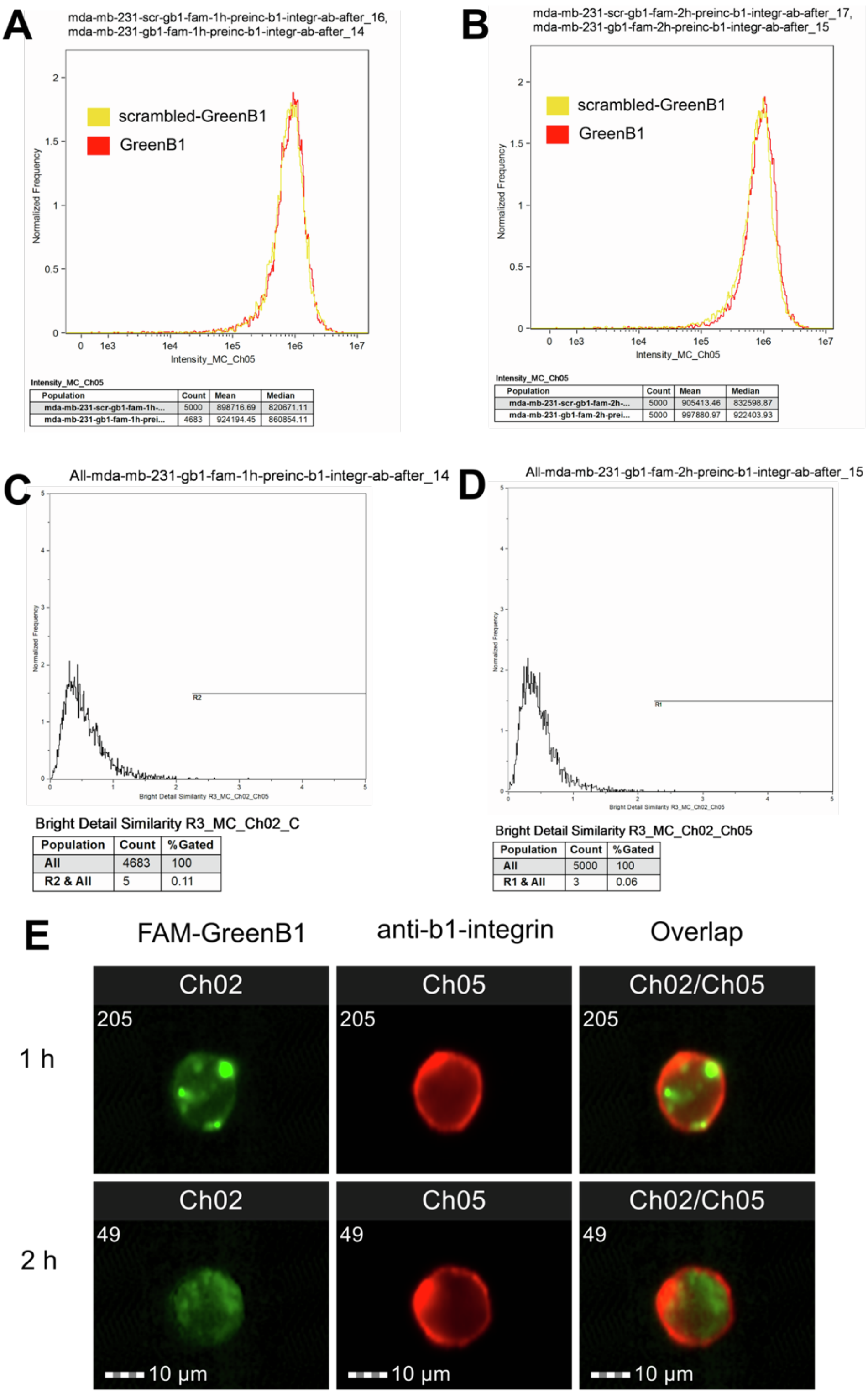

Figure S7. The co-localization of pre-incubated FAM-GreenB1 and anti-β1-integrin antibody on MDA-MB-231 cells. The amount of anti-β1-integrin-PE-Cy5 antibody binding to MDA-MB-231 cells was not affected after pre-incubation with FAM-GreenB1 or FAM-scrambled-GreenB1 for either 1 hour (A) or 2 hours (B). Only a small fraction of cells, 0.11% after 1 hour (C) and 0.06% after 2 hours (D) were classified as co-localization events between

FAM-GreenB1 and anti-β1-integrin-PE-Cy5 antibody. Representative images of FAM-GreenB1 and the antibody are shown, with the representative cells having a Bright Detail Similarity value equal to the mode of the histogram (E).

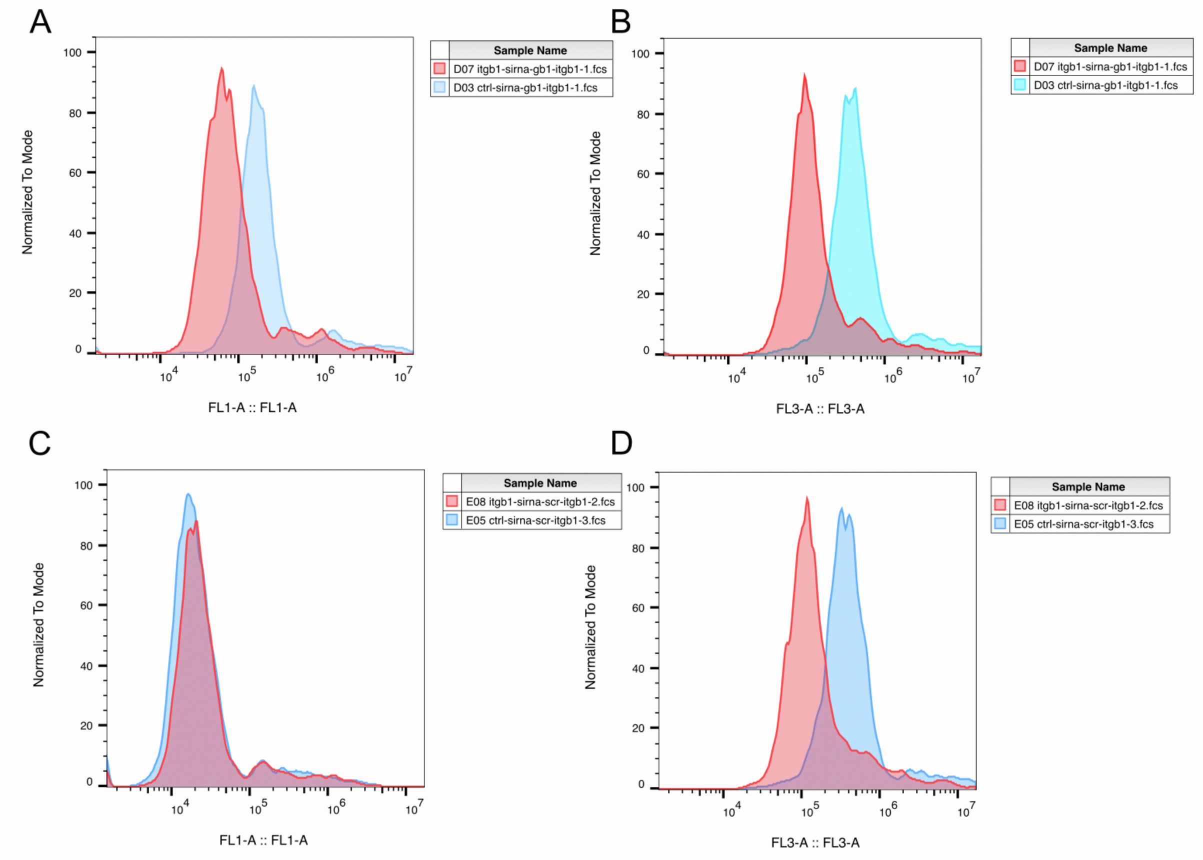

Figure S8. Representative chromatograms of reduced GreenB1 binding after siRNA induced β1-integrin expression decrease in MDA-MB-231 cells. FAM-GreenB1 binding is reduced upon β1-integrin silencing (red) compared to when control siRNA is used (blue) (A) and anti-β1-integrin antibody binding is also reduced to similar extent when β1-integrin siRNA (red) is used instead of control siRNA (blue) (B). FAM-scrambled-GreenB1 binding is not affected by transfection of β1-integrin siRNA (red) or control siRNA (blue) (C) while anti-β1-integrin antibody binding is reduced when β1-integrin siRNA (red) is used instead of control siRNA (blue) (D).

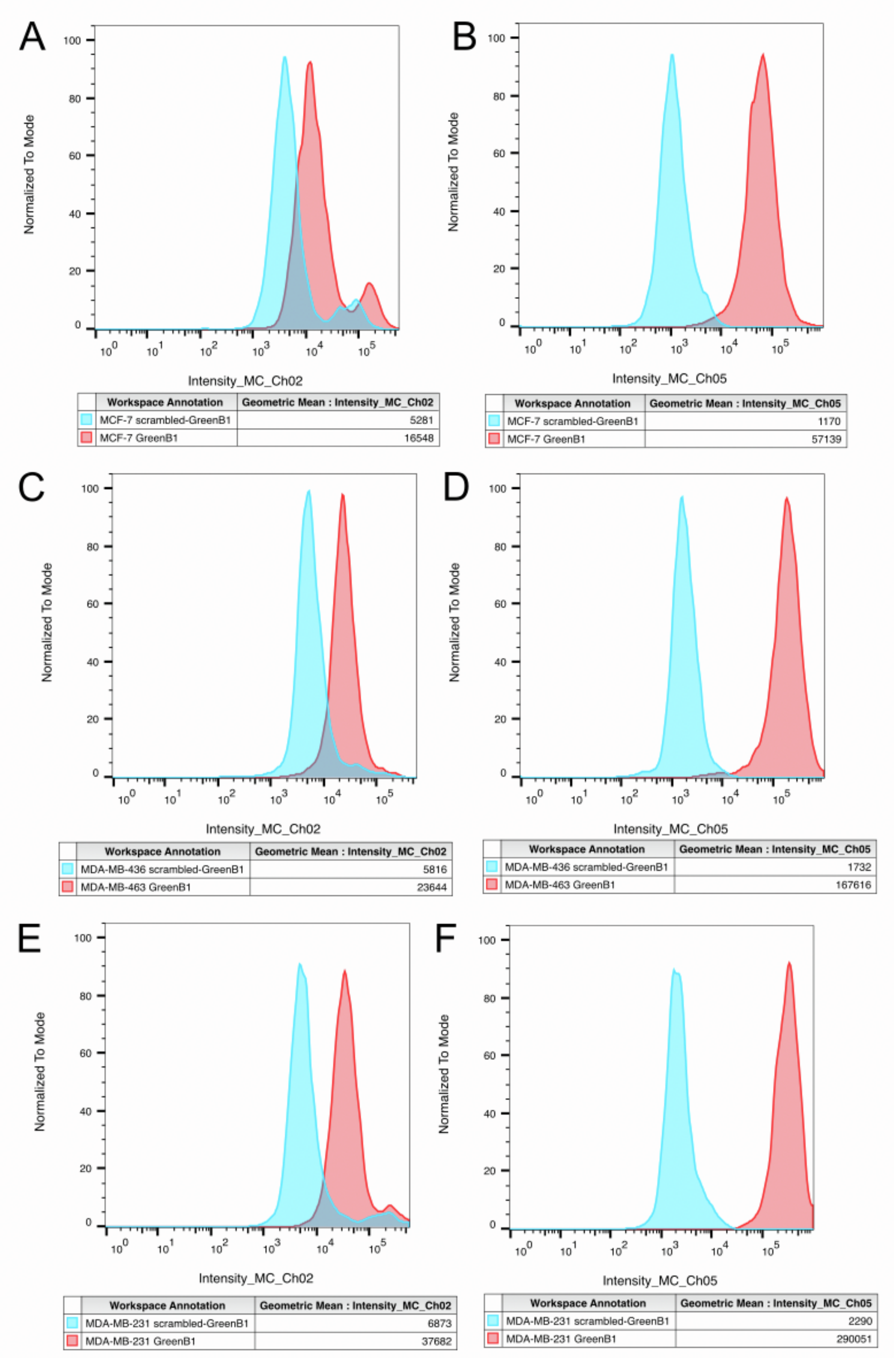

Figure S9. Representative chromatograms of FAM-GreenB1 and PE-Cy5 anti-β1-integrin antibody binding correlation. GreenB1 binding (red) and scrambled-GreenB1 (blue) (A) and isotype control antibody (blue) and anti-β1-integrin antibody (red) (B) binding to MCF-7 cells. GreenB1 binding (red) and scrambled-GreenB1 (blue) (C) and isotype control antibody (blue) and anti-β1-integrin antibody (red) (D) binding to MDA-MB-436 cells. GreenB1 binding (red) and scrambled-GreenB1 (blue) (E) and isotype control antibody (blue) and anti-β1-integrin antibody (red) (F) binding to MDA-MB-231 cells.

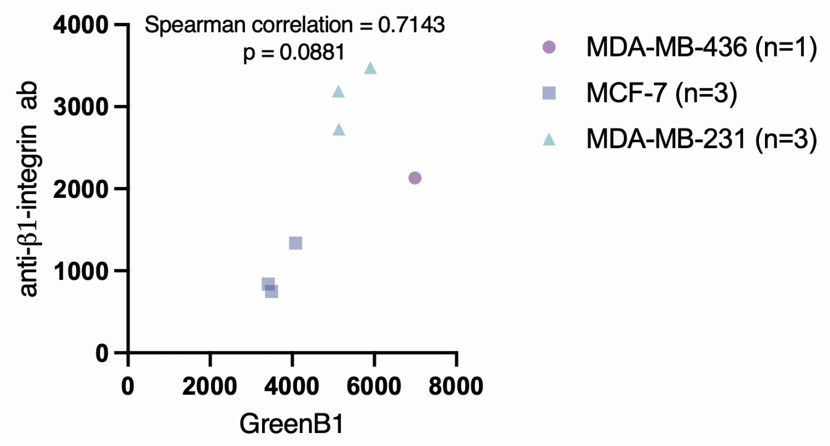

Figure S10. Correlation of scrambled-GreenB1 binding and isotype control antibody is not statistically significant (Spearman correlation = 0.7143, p = 0.0881) on MDA-MB-231, MCF-7 and MDA-MB-436 cell lines.
